# The mechanism behind the selection of two different cleavage sites in NAG-NAM polymers

**DOI:** 10.1107/S2052252517000367

**Published:** 2017-02-23

**Authors:** Marko Mihelič, Kristina Vlahoviček-Kahlina, Miha Renko, Stephane Mesnage, Andreja Doberšek, Ajda Taler-Verčič, Andreja Jakas, Dušan Turk

**Affiliations:** a Department of Biochemistry and Molecular and Structural Biology, Jozef Stefan Institute, Jamova 39, 1000 Ljubljana, Slovenia; b Centre of Excellence for Integrated Approaches in Chemistry and Biology of Proteins, Jamova 39, 1000 Ljubljana, Slovenia; c Division of Organic Chemistry, Rudjer Boskovic Institute, Bijenicka cesta 54, 10000 Zagreb, Croatia; d Krebs Institute, University of Sheffield, Firth Court, Western Bank, Sheffield S10 2TN, England

**Keywords:** *Staphylococcus aureus*, autolysins, substrate specificity, *N*-acetylglucosaminidase, muramidases, lysozyme

## Abstract

Peptidoglycan is a giant molecule that forms the cell wall that surrounds bacterial cells. It is composed of alternating *N*-acetylglucosamine (NAG) and *N*-acetylmuramic acid (NAM) residues connected by β-(1,4)-glycosidic bonds and cross-linked with short polypeptide chains. Owing to the increasing antibiotic resistance against drugs targeting peptidoglycan synthesis, studies of enzymes involved in the degradation of peptidoglycan, such as *N*-acetylglucos­aminidases, may expose new, valuable drug targets. The scientific challenge addressed here is how lysozymes, muramidases which are likely to be the most studied enzymes ever, and bacterial *N*-acetylglucosaminidases discriminate between two glycosidic bonds that are different in sequence yet chemically equivalent in the same NAG-NAM polymers. In spite of more than fifty years of structural studies of lysozyme, it is still not known how the enzyme selects the bond to be cleaved. Using macromolecular crystallography, chemical synthesis and molecular modelling, this study explains how these two groups of enzymes based on an equivalent structural core exhibit a difference in selectivity. The crystal structures of *Staphylococcus aureus* *N*-acetylglucosaminidase autolysin E (AtlE) alone and in complex with fragments of peptidoglycan revealed that *N*-acetylglucosaminidases and muramidases approach the substrate at alternate glycosidic bond positions from opposite sides. The recognition pocket for NAM residues in the active site of *N*-acetylglucosaminidases may make them a suitable drug target.

## Abstract 2

The crystal structure of autolysin E, an *N*-acetylglucosaminidase from *S. aureus*, reveals the structural basis of the selection mechanism of muramidases and glucosaminidases for docking the two chemically equivalent, yet distinct in sequence, β-glycosidic bonds in NAG-NAM polymers.

## 1. Introduction   

Peptidoglycan (PG) is a living structure that builds the bacterial cell wall. Bacterial growth, division, colonization and biofilm formation rely heavily on the ability of cells to remodel their wall, which includes both the degradation and synthesis of PG. Staphylococci represent a large group of bacteria that live on humans and can cause severe infections in immunocompromised people (Lowy, 1998 ▸; Varrone *et al.*, 2011 ▸; Vincent *et al.*, 2009 ▸). The widespread use of antibiotics in recent decades has resulted in the emergence of antibiotic-resistant and multiple antibiotic-resistant strains such as β-lactam-antibiotic (penicillin) resistant [extended-spectrum β-lactamase (ESBL)-producing] bacteria, methicillin-resistant *Staphylococcus aureus* (*S. aureus*) (MRSA) and vancomycin-resistant *S. aureus* (VRSA) (Archer, 1998 ▸; Dantes *et al.*, 2013 ▸; Gardete & Tomasz, 2014 ▸; Hanberger *et al.*, 2011 ▸; Hiramatsu *et al.*, 1997 ▸; Nunes *et al.*, 2007 ▸; Xia *et al.*, 2013 ▸; Zetola *et al.*, 2005 ▸). Therefore, it is important to explore alternative targets for the treatment of bacterial infections.

Alternating *N*-acetylglucosamine (NAG) and *N*-acetyl­muramic acid (NAM) residues connected by β-(1,4)-glycosidic bonds and cross-linked with short polypeptide chains assemble the PG (Vocadlo *et al.*, 2001 ▸). Two groups of enzymes, muramidases and *N*-acetylglucosaminidases, cleave alternate but chemically equivalent glycosidic bonds in the NAG-NAM polymers.

Lysozyme, a muramidase, is the first line of immune defence against bacteria. It was the first enzyme and the second protein for which a crystal structure was determined at atomic resolution (Blake *et al.*, 1965 ▸; Johnson, 1998 ▸) and is likely to be the most studied enzyme ever. Research to date has addressed its folding, evolution and catalytic mechanism (Anderson *et al.*, 1981 ▸; Kuroki *et al.*, 1993 ▸; Matthews, 1996 ▸; Matthews *et al.*, 1981 ▸; Vocadlo *et al.*, 2001 ▸); however, insight into the mechanism of the selectivity of its binding of NAG-NAM polymers has remained unexplored. We still do not know how lysozymes differ from *N*-acetylglucosaminidases in terms of recognition of the scissile bond. Yet, the substrate selectivity among hydrolases of different kinds and species is the foundation of the differences in their biological roles.

The *S. aureus* Mu50 genome (an MRSA strain with vancomycin-intermediate resistance; VISA) encodes five *N*-acetyl­glucosaminidases belonging to glycoside hydrolase family 73 (GH73 family). Four of them, SAV2307, SAV1052, SAV1775 and SAV2644 [also named AtlE (SagA, LytD), AtlA, SagB and ScaH, respectively; SAVxxxx identifiers are according to entries in the http://www.genome.jp database], are widely distributed throughout the genomes of *S. aureus* strains and are critical for cell enlargement. It has been shown that *S. aureus* lacking all five *N*-acetylglucosaminidases is not viable, which implies that they are essential for cell viability (Wheeler *et al.*, 2015 ▸). The best studied among them is SAV1052, the major bifunctional autolysin (AtlA; Biswas *et al.*, 2006 ▸; Heilmann *et al.*, 1997 ▸; Oshida *et al.*, 1995 ▸; Sugai *et al.*, 1995 ▸).

The amidase activity of AtlA was confirmed and analyzed by structural studies of homologous enzymes from *S. epidermidis* (Zoll *et al.*, 2010 ▸) and later *S. aureus* (Büttner *et al.*, 2014 ▸). However, the other *N*-acetylglucosaminidases from *S. aureus* remained unexplored. The crystal structure of AtlE and its complexes with substrate fragments described here provide insight into the mechanism of NAG-NAM binding, whereas a comparison with structural data for lysozymes and their complexes with substrate fragments enabled us to seek out the structural differences responsible for docking of the two alternate glycosidic bonds in the NAG-NAM polymer.

## 2. Methods   

### 2.1. Cloning, protein production and purification   

**Figure 1:**
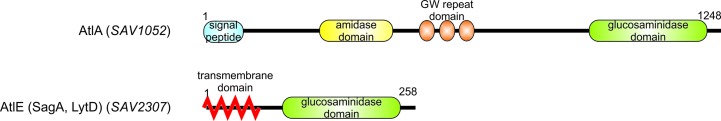
Domain organization of *S. aureus* autolysins AtlA and AtlE. Proteins are marked with the protein and the gene name. Only the *N*-acetylglucosaminidase domains were used in this study.

AtlE is a 258-amino-acid protein encoded by the SAV2307 gene in the *S. aureus* Mu50 genome, while AtlA is a 1248-amino-acid protein encoded by the SAV1052 gene from the same genome (Fig. 1 ▸). The truncated sequences of the glucosaminidase domain of AtlE (SAV2307 residues 35–258; UniProt code A0A0H3JT72) and the glucosaminidase domain of AtlA (Glu-AtlA; SAV1052 residues 1012–1231; UniProt code Q931U5) were used. The nucleotide sequences were amplified from the genomic DNA of *S. aureus* Mu50 using KOD Hot Start Polymerase and were cloned into the pMCSG7 plasmid in frame with an N-terminal His tag as described by Eschenfeldt *et al.* (2009 ▸). The mutants were prepared by the overlap extension method (Ho *et al.*, 1989 ▸).

The genes were expressed in the *Escherichia coli* (*E. coli*) BL21 (DE3) expression strain grown in ZYM5052 autoinduction medium (Studier, 2005 ▸). To facilitate production of the protein in a soluble form, the cells were initially grown at 37°C. When the optical density measured at 600 nm (OD_600_) reached 1, the cells were transferred to 25°C and left for 16 h.

Selenomethionine minimal medium (SeMetMM) was prepared as described by Guerrero *et al.* (2001 ▸). A culture of the *E. coli* BL21 (DE3) pMCSG7-AtlE transformants was grown overnight in 20 ml LB medium supplemented with ampicillin (100 µg ml^−1^) at 37°C with shaking at 250 rev min^−1^. The next day, this cell suspension was used as an inoculum for 1 l of the same medium and the OD_600_ was monitored until it reached 1. The cell culture was then centrifuged for 15 min at 4000 rev min^−1^ and the pellet was resuspended in 1 l SeMetMM with a final concentration of 1 m*M* IPTG and incubated at 18°C and 250 rev min^−1^ for an additional 20 h.

The cells were pelleted by centrifugation (15 min at 7000*g*), resuspended in buffer *A* (0.03 *M* Tris pH 7.5, 0.4 *M* NaCl) supplemented with 1 mg ml^−1^ lysozyme, and frozen and disrupted by freeze–thaw cycles and sonication. The proteins were purified from the cell lysate on an ÄKTAxpress FPLC system (GE Healthcare) using a two-step purification protocol. The first purification step was Ni^2+^-affinity chromatography on a HiTrap IMAC FF column (GE Healthcare) equilibrated in buffer *A* with 10 m*M* imidazole. The bound proteins were eluted with buffer *A* containing 300 m*M* imidazole and applied onto a HiPrep 26/60 Sephacryl S200 size-exclusion column (GE Healthcare) equilibrated in buffer *A*. The fractions containing the pure protein were collected, concentrated, desalted against 20 m*M* HEPES pH 7.5, 100 m*M* NaCl and stored at −20°C.

### 2.2. Biochemical analysis of AtlE and Glu-AtlA activities   

**Figure 2:**
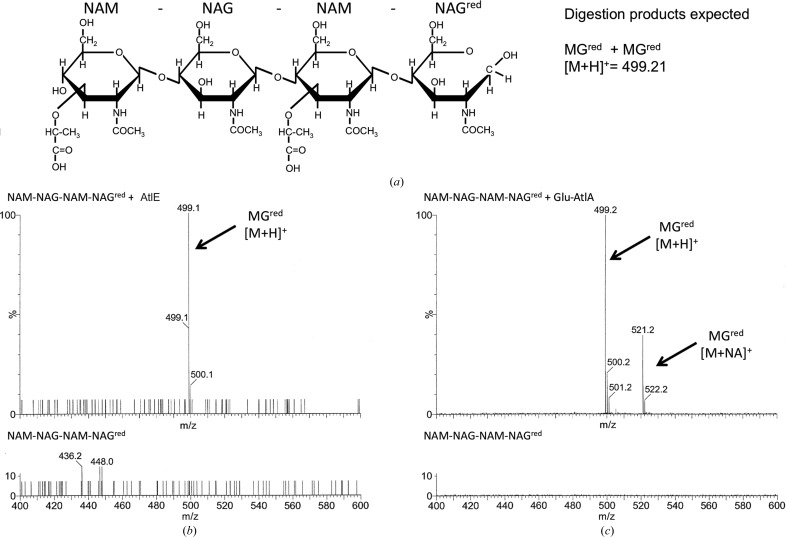
Analysis of AtlE and Glu-AtlA digestion products of (NAM-NAG)_2_^red^ substrate. (*a*) Schematic representation of the tetrasaccharide substrate with the expected digestion products. (*b*, *c*) Mass-spectrometric analysis of the digestion products of (*b*) AtlE and (*c*) Glu-AtlA. The expected molecular peaks are annotated. The same amount of substrate/digestion products was analysed in both cases.

AtlE and Glu-AtlA were tested against the *S. aureus* cell wall (Odintsov *et al.*, 2004 ▸) and two synthetic substrates: the (NAM-NAG)_2_^red^ tetrasaccharide (Fig. 2 ▸) and (NAG)_6_^red^. Analysis of the degradation products was performed by mass spectrometry.

The tetrasaccharide (NAM-NAG)_2_ was purified by HPLC using *Micrococcus lysodeikticus* peptidoglycan fragments solubilized by digestion with *Enterococcus faecalis* autolysin A. (NAG)_6_ was purchased from Dextra Laboratories.

### 2.3. NAG-NAM disaccharide synthesis   

**Figure 3:**
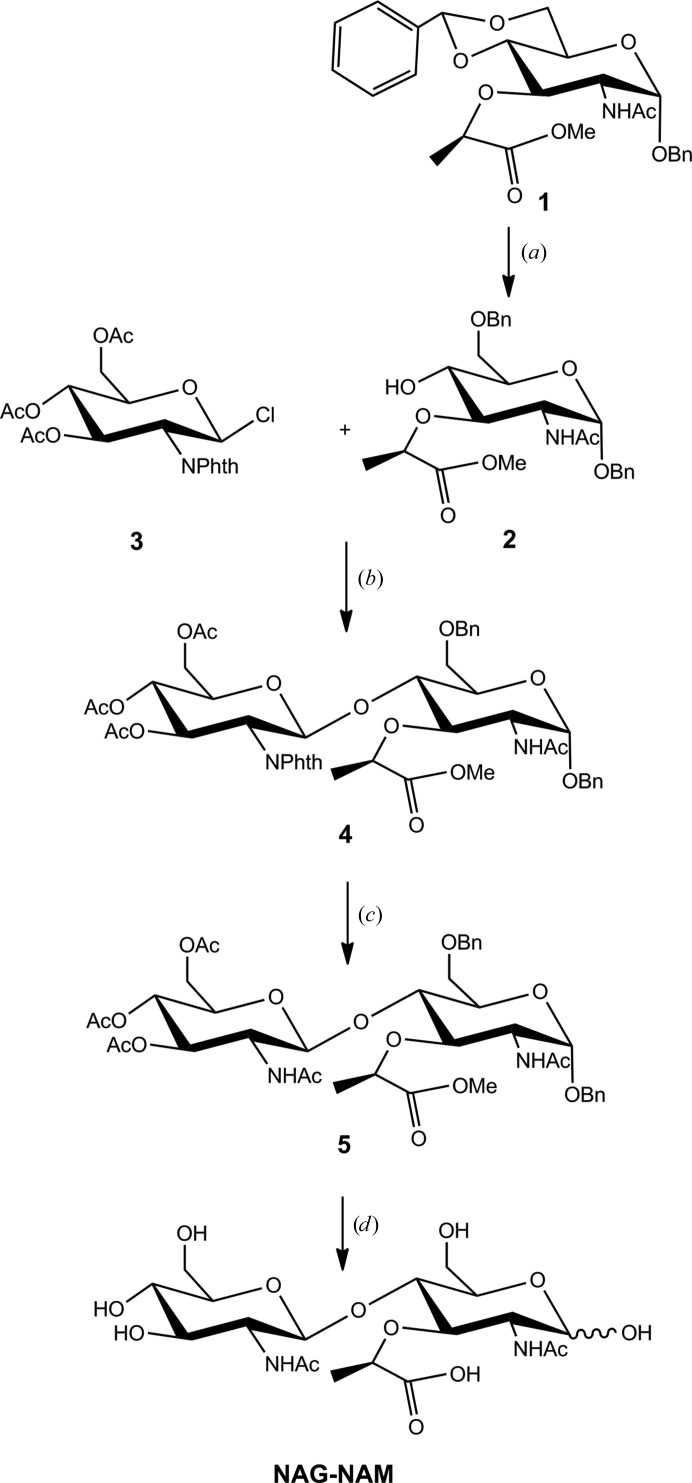
Synthesis of the NAG-NAM disaccharide. Reagents and conditions: (*a*) Et_3_SiH, I_2_, 0°C, 2 h; (*b*) AgTf, RT, 18 h; (*c*) NaOMe, MeOH, RT, 1 h; hydrazine hydrate, EtOH, 80°C, 2 h; pyridine, acetic anhydride, RT, 18 h; (*d*) 0.5 *M* KOH, dioxane, RT, 48 h; H_2_, Pd/C, EtOH:HOAc:water; RT, 18 h.

The NAG-NAM disaccharide {2-acetamido-4-*O*-(2-acetamido-2-deoxy-β-d-glucopyranosyl)-3-*O*-[(*R*)-1-carboxyethyl]-2-deoxy-α-d-glucopyranose} was prepared according the protocol introduced by Kantoci *et al.* (1987 ▸) and papers cited therein, with some revisions (Fig. 3 ▸). Selective opening of the 4,6-benzylidene ring of benzyl 2-acetamido-4,6-*O*-benzylidene-3-*O*-[(*R*)-1-(methoxycarbonyl)ethyl]-2-deoxy-α-d-glucopyranoside (**1**) to give benzyl 2-acetamido-6-*O*-benzyl-3-*O*-[(*R*)-1-(methoxycarbonyl)­ethyl]-2-deoxy-α-d-glucopyranoside (**2**) was performed with iodine and triethylsilane instead of sodium cyanoborohydride as previously described (Keglevic *et al.*, 1985 ▸). Glycosidic bond formation between activated glucosamine 3,4,6-tri-*O*-acetyl-2-deoxy-2-phthalimido-β-d-glucopyranosyl chloride (**3**) and selectively protected muramic acid **2** in the presence of silver trifluoromethanesulfonate in extremely dry conditions gave 2-acetamido-4-*O*-(3,4,6-tri-*O*-acetyl-2-deoxy-2-phthalimido-β-d-glucopyranosyl)-6-*O*-benzyl-2-deoxy-3-*O*-[(*R*)-1-methoxycarbonyl)ethyl]-α-d-glucopyranoside (**4**). Removal of the phthalimido group from compound **4** with hydrazine followed by acetylation gave benzyl 2-acetamido-4-*O*-(2-acetamido-3,4,6-tri-*O*-acetyl-2-deoxy-β-d-gluco­pyranosyl)-6-*O*-benzyl-2-deoxy-3-*O*-[(*R*)-1-(methoxycarbonyl)ethyl]-α-d-glucopyranoside (**5**). Saponification of the acetyl and methyl groups and removal of the benzyl groups with catalytic hydrogenation gave NAG-NAM (Kantoci *et al.*, 1987 ▸; Keglevic *et al.*, 1985 ▸).

#### 2.3.1. Benzyl 2-acetamido-6-*O*-benzyl-3-*O*-[(*R*)-1-(methoxycarbonyl)ethyl]-2-deoxy-α-d-glucopyranoside (**2**)   

Compound **1** (630 mg; 1.3 mmol) was dissolved in dry dichloromethane (DCM; 10 ml), and iodine (370 mg) and Et_3_SiH (3.7 ml) were added. The reaction was stirred in an ice bath, and after 30 min and 1 h additional iodine (37 mg) and Et_3_SiH (370 µl) were added. The reaction was terminated after 2 h, diluted with DCM (40 ml) and washed first with NaHCO_3_ (20 ml) and then with water (20 ml). The organic layers were dried with Na_2_SO_4_, evaporated and chromatographed on a silica-gel column in 3:2 DCM:acetone and 9:1 DCM:methanol (MeOH) solvent systems. Crystallization from acetone:diisopropyl ether gave compound **2** (330 mg; 52%).

Electrospray ionization mass spectrometry (ESI-MS): C_26_H_33_NO_8_, 488.4 [*M*+H]^+^; calculated, 488.5. Rf = 0.65 (9:1 DCM:MeOH).

#### 2.3.2. Benzyl 2-acetamido-4-*O*-(3,4,6-tri-*O*-acetyl-2-deoxy-2-phthalimido-β-d-glucopyranosyl)-6-*O*-benzyl-2-deoxy 3-O-[(*R*)-1-(methoxycarbonyl)ethyl]-α-d-glucopyranoside (**4**)   

The glucosyl chloride **3** (280 mg; 0.62 mmol) and protected muramic acid **2** (100 mg; 0.21 mmol) with silver trifluoro­methanesulfonate (AgTf; 210 mg; 0.82 mmol) as a catalyst were subjected to Anderson’s apparatus for glycosidic coupling (Nashed & Anderson, 1982 ▸) followed by molecular sieving. Dry DCM (2 ml) was added and the reaction was stirred overnight under nitrogen at room temperature. After this, chloroform was added to the suspension and it was centrifuged. The residue was washed twice with chloroform. The chloroform supernatants were washed with a saturated aqueous solution of NaHCO_3_ and then with water, and then dried over Na_2_SO_4_. The solvent was evaporated and the product was purified by flash silica-gel column chromatography in 8:4:1 diethyl ether:petroleum ether:isopropanol (iPrOH) and 9:1 DCM:MeOH solvent systems. After the second column, compound **4** (62 mg; 33%) was obtained.

ESI-MS: C_46_H_53_N_2_O_17_, 905.4 [*M*+H]^+^; calculated, 905.3; C_46_H_52_N_2_NaO_17_, 927.4 [*M*+Na]^+^; calculated, 927.3. Rf = 0.54 (8:4:1 diethyl ether:petroleum ether:isopropanol).

#### 2.3.3. Benzyl 2-acetamido-4-*O*-(2-acetamido-3,4,6-tri-*O*-acetyl-2-deoxy-β-d-glucopyranosyl)-6-*O*-benzyl-2-deoxy-3-*O*-[(*R*)-1-(methoxycarbonyl)ethyl]-α-d-glucopyranoside (**5**)   

Disaccharide 4 (45 mg; 0.0498 mmol) was dissolved in dry MeOH (1.376 ml) with 0.1 *M* NaOMe/MeOH (145 µl). The reaction was stirred at room temperature for 1 h, after which additional 0.1 *M* NaOMe/MeOH (145 µl) was added and stirring was continued for 15 min. The reaction solution was neutralized with Amberlite IR-120 (H^+^), filtered and evaporated. The residue was dissolved in 96% ethanol (2.25 ml) and hydrazine hydrate (16.88 µl). The reaction was stirred for 2 h under reflux (80°C). The reaction mixture was evaporated after the addition of toluene. The residue was dissolved in 1:1 pyridine:acetic anhydride (1.2 ml) and stirred overnight. After this, the solvent was evaporated after the addition of toluene, and the residue was purified by flash silica-gel column chromatography in 2:3:1 ethyl acetate (EtOAc):iPrOH:petroleum ether to give compound **5** (27 mg; 67%).

ESI-MS: C_40_H_52_N_2_NaO_16_, 839.3 [*M*+Na]^+^; calculated, 839.3. Rf = 0.50 (2:3:1 EtOAc:iPrOH:petroleum ether).

#### 2.3.4. 2-Acetamido-4-*O*-(2-acetamido-2-deoxy-β-d-glucopyranosyl)-3-*O*-[(*R*)-1-carboxyethyl]-2-deoxy-α-d-glucopyran­ose (NAG-NAM)   

Compound **5** (35 mg; 0.043 mmol) was dissolved in dioxane (1.75 ml) and 0.5 *M* KOH (0.875 ml) was added to adjust the pH to 12. The reaction was stirred at room temperature for 48 h and then neutralized by Amberlite IR-120 (H^+^), filtered and evaporated. The residue was dissolved in 6:1.5:1.5 EtOH:acetic acid (HOAc):water (5.25 ml), and Pd/C (10%; 46 mg) was added. The reaction was hydrogenated at room temperature overnight. After this, the reaction was filtered over a small column of Celite to remove the catalyst, and the filtrate was evaporated. The residue was crystallized from 1:10 MeOH:ether to give NAG-NAM (15 mg; 70%).

ESI-MS: C_19_H_32_N_2_NaO_13_, 519.2 [*M*+Na]^+^; calculated, 519.2. Rf = 0.55 (1:1:1:1 *n*-butanol:HOAc:EtOAc:water).

### 2.4. Protein crystallization and structure determination   

Crystals of AtlE (concentrated to 15 mg ml^−1^ in 20 m*M* HEPES pH 7.5, 100 m*M* NaCl) were grown in 2 *M* NaCl, 2 *M* ammonium sulfate using the vapour-diffusion method. The crystallization drop consisted of 1 µl protein solution and 1 µl crystallization buffer. The crystals were cryoprotected by soaking in the crystallization buffer containing 30% glycerol. Data were collected from native and SeMet-derivative crystals on the XRD beamline at the Elettra synchrotron, Trieste, Italy.

The native structure was solved with the help of an SeMet derivative using data collected at the remote wavelength, exploiting the anomalous signal from seven SeMet residues using the *HKL*-3000 software (Minor *et al.*, 2006 ▸). The native structure was rebuilt and refined using *MAIN* (Turk, 2013 ▸) and *REFMAC* (Murshudov *et al.*, 2011 ▸), and was deposited in the PDB (Berman *et al.*, 2000 ▸) with accession code 4pia.

### 2.5. Determination of the crystal structures of peptidoglycan-fragment complexes   

The NAG-NAM disaccharide was synthesized as described and muramyl dipeptide (MDP) was purchased from Invitrogen. Complexes with AtlE with NAG-NAM and MDP were obtained by soaking native crystals with a 10 m*M* solution of the ligands. Data from crystals of the native protein complexed with MDP and of the E138A mutant complexed with NAG-NAM were collected at a wavelength of 0.9184 Å on beamline 14.1 at the BESSY synchrotron, Berlin, Germany, whereas the diffraction data for the native enzyme complexed with NAG-NAM were collected at an in-house X-ray source using a copper rotating anode (Bruker). The diffraction data were integrated with *HKL*-2000 (Otwinowski & Minor, 1997 ▸). The structures were built with *MAIN* (Turk, 2013 ▸) using the topology library and geometric restraints provided by PURY (Andrejašič *et al.*, 2008 ▸) and finally refined with *REFMAC* (Murshudov *et al.*, 2011 ▸) for deposition. The geometry of the binding of the disaccharide was equivalent in the two crystal structures; therefore, only the complex with the native sequence is shown in the figures. However, all three crystal structures were deposited in the PDB with accession codes 4pi7, 4pi8 and 4pi9. For data and refinement statistics, see Table 1 ▸.

### 2.6. Similarity to other structures   

The *DALI* server (Holm & Rosenström, 2010 ▸) was used to identify glucosaminidase enzymes belonging to the GH73 family in the CAZy database (Lombard *et al.*, 2014 ▸) with similar structures to the determined structure of AtlE. The identified proteins were analyzed by structure-based sequence alignment performed by *STRAP* (Gille & Frömmel, 2001 ▸).

### 2.7. Superimposition of substrate fragments on the AtlE complexes   

To compare the binding of the NAG-NAM disaccharide and MPD in the light of the structures of the other related complexes, we superimposed both AtlE complexes with the crystal structures of NAG_3_ bound to goose-type lysozyme from Atlantic cod (ACOD; Helland *et al.*, 2009 ▸; PDB entry 3gxr) as the closest related lysozyme, NAG-NAM-peptide bound to T4 lysozyme (T4_L; Kuroki *et al.*, 1993 ▸; PDB entry 148l), NAG_3_ bound to goose lysozyme (GLYZ; Weaver *et al.*, 1995 ▸; PDB entry 154l) and NAM-NAG-NAM in complex with chicken lysozyme (CLYZ; Kelly *et al.*, 1979 ▸; PDB entry 9lyz) (see Fig. 7*c*).

### 2.8. Molecular modelling of NAG-NAM substrates into the active sites of AtlE and ACOD   

The (NAG)_6_ was built first. The model was built by filling the gap between the (NAG)_2_ and (NAG)_3_ parts of the ACOD structure (Helland *et al.*, 2009 ▸; PDB entry 3gxr) with the missing NAG residue. The resulting hexasaccharide was energetically minimized by restraining the matching saccharide residues to the crystal structures of the ACOD complex using *MAIN* (Turk, 2013 ▸). To build a model that matches the muramidase/lysozyme substrate, the corresponding NAG residues were substituted with NAM residues. The resulting (NAG-NAM)_3_ hexasaccharide was energetically minimized again.

To build the substrate model corresponding to *N*-acetyl­glucosaminidase activity, the structures of AtlE and ACOD were superimposed using *FATCAT* (Ye & Godzik, 2003 ▸). Using superimposition parameters, the (NAG)_6_ model was transferred from the ACOD environment to the AtlE structure. Then, similarly as for lysozyme, the corresponding alternate NAG residues were substituted with NAM residues. The resulting (NAG-NAM)_3_ model was slightly shifted to match the position of the NAG-NAM disaccharide in the complex with AtlE, and was energetically minimized by constraining the positions of the atoms of the −3, −2 residues to the positions observed in the crystal structure.

## 3. Results   

### 3.1. Biochemical activity   

Both proteins, AtlE and Glu-AtlA, were active against the cell-wall substrate, and they both only cleaved the NAG-β-(1,4)-NAM glycosidic bond, which corresponds to *N*-acetylglucos­aminidase activity, whereas (NAG)_6_ was not cleaved. The cleavage of the (NAM-NAG)_2_^red^ tetrasaccharide producing the NAM-NAG and NAM-NAG^red^ di­saccharides indicates *N*-acetylglucos­aminidase activity, whereas NAM^red^ and NAG-NAM-NAG^red^ products would indicate muramidase activity. Only NAM-NAG^red^, with a molecular weight of 499.21 Da, was found in both cases (Fig. 2 ▸).

### 3.2. Structure   

**Figure 4:**
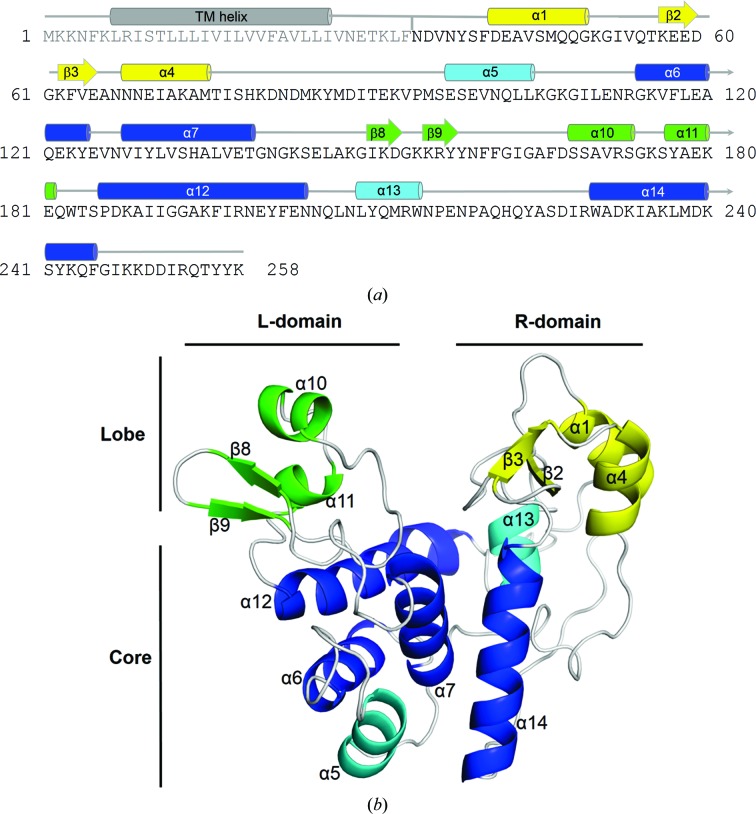
Structure of AtlE. The fold of the structure in the core and lobe regions is shown. The compact α-­helical structural core forms the lower part of both domains, whereas the R- and L-lobes containing short α-­helical and β-strand regions reside on top of the R- and L-domains, respectively. Between the lobes, there is a long groove that runs across the entire central part of the molecule. The secondary-structure elements in the figures are numbered in the order that they occur in the sequence. The core of the structure consists of six helices of different lengths: α5, α6, α7 and α12 from the L-domain and α13 and α14 from the R-domain. In contrast, each lobe contains two short α-helices and two β-­hairpins. In the L-lobe the β-­hairpin precedes the two α-helices, whereas in the R-lobe the β-hairpin is positioned between them (α1 and α4). (*a*) Sequence of AtlE. The grey text indicates the part excluded from expression. The regions corresponding to the secondary-structure elements are shown in the same colour code as used in (*b*). (*b*) Fold of AtlE. The four conserved helices in the core region are coloured blue, whereas the other two core region helices are shown in cyan. The secondary-structure elements belonging to the L- and R-lobes are shown in green and yellow, respectively.

AtlE is well defined along the whole chain apart from the first three residues, which were built as alanines, and His79 and Lys80, which were disordered and therefore were not built (Fig. 4 ▸ *a*). Several side chains were built with alternative conformations and a few side chains were disordered. Helix α10 has higher *B* values, most likely indicating statistical disorder of this surface feature. The AtlE crystal structure (Table 1 ▸) shows a heart-shaped globular fold composed of separate left (L) and right (R) domains (Fig. 4 ▸ *b*). To address the structural parts, we further divided the structure into core and lobe regions. The compact α-helical structural core forms the lower part of both domains, whereas the R-lobe and L-lobe contain short α-helical and β-strand regions, respectively. Between the lobes, there is a long groove that runs across the entire central part of the molecule. The structure has additional five amino acids (SNAAA) at the N-terminus remaining after cleavage of the His tag by TEV protease. The secondary-structure assignment of the AtlE structure (Figs. 4 ▸ and 5 ▸) is used as the reference in comparisons with other structures.

### 3.3. Similarity to other structures   

**Figure 5:**
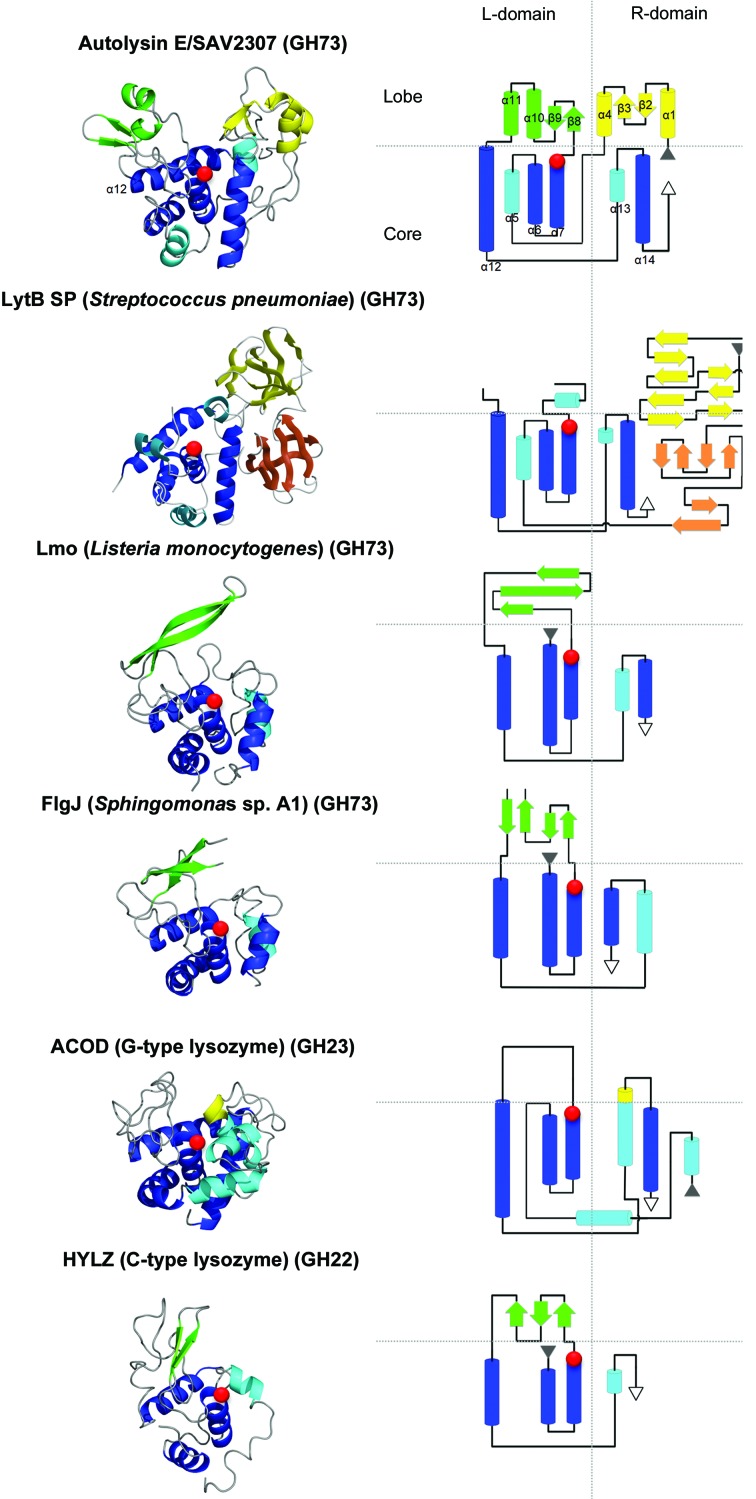
Structural similarity of AtlE. AtlE and the related structures of LytB SP (PDB entry 4q2w), Lmo (PDB entry 3fi7), FlgJ (PDB entry 2zyc), ACOD (PDB entry 3gxk) and HLYZ (PDB entry 1iwt) are presented from top to bottom. The left column shows the chain trace with the secondary-structure elements in the same orientation. The right column presents the architecture of the folds schematically. Helices are shown as cylinders and β-strands as arrows. The colour codes are the same as those used in Fig. 4 ▸. Red circles mark the position of the catalytic glutamic acid. Three-dimensional images of folds were prepared with *PyMOL* (DeLano, 2002 ▸) and *MAIN* (Turk, 2013 ▸) and were rendered with *Raster*3*D* (Merritt & Bacon, 1997 ▸).

**Figure 6:**
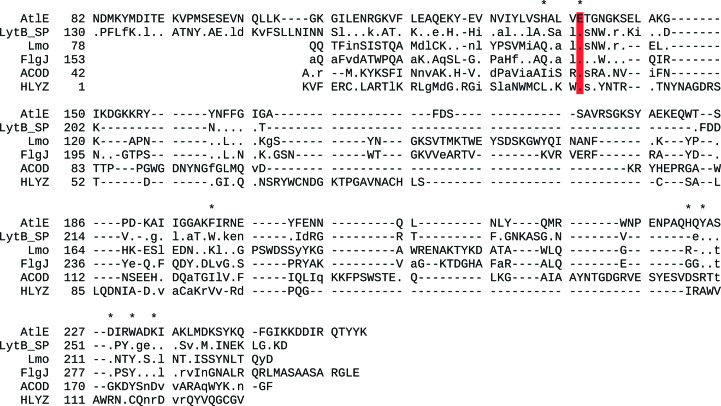
Structure-based sequence alignment of AtlE, LytB SP, ACOD, Lmo, FlgJ and HLYZ (the proteins from Fig. 5 ▸). Alignment was performed with *STRAP* (Gille & Frömmel, 2001 ▸). The regions at the N-termini, which do not exhibit any similarity among the structures, were excluded from this alignment. Hyphens correspond to deletions, whereas dots, lowercase and uppercase characters correspond to residues that are identical, similar and different, respectively, from the sequence at the top. The catalytic Glu residue and the residues addressed in the text for their importance in substrate binding are marked with asterisks.

Using the crystal structure of AtlE, the *DALI* server identified several glucosaminidase enzymes with similar structures belonging to the GH73 family in the CAZy database (Lombard *et al.*, 2014 ▸): *Streptococcus pneumoniae* LytB (LytB SP; PDB entry 4q2w; *Z*-score = 16.9; Bai *et al.*, 2014 ▸), *Listeria monocytogenes* Lmo1076 (Lmo; PDB entry 3fi7; *Z*-score = 8.0; Bublitz *et al.*, 2009 ▸) and *Sphingomonas* sp. A1 FlgJ (PDB entry 2zyc; *Z*-score = 7.3; Hashimoto *et al.*, 2009 ▸). A significant structural homology was also found with the G-type lysozyme from Atlantic cod (ACOD; PDB entry 3gxr; *Z*-score = 6.7; Helland *et al.*, 2009 ▸) belonging to the GH23 family. Owing to the similarity in architecture, although not spotted by the *DALI* server, we also included the human lysozyme structure (HLYZ; PDB entry: 1iwt) in the comparison as a representative of the goose-type lysozymes (Joti *et al.*, 2002 ▸). The alignment of the six enzymes in Figs. 5 ▸ and 6 ▸ reflects their structural similarity, but also points out their diversity. This is also reflected in the rather large root-mean-square deviation (r.m.s.d.) of superimposed structures, which is in the range from 1.9 to 5.0 Å (Table 2 ▸). The structure-based alignment of all sequences (Fig. 6 ▸) by *STRAP* (Gille & Frömmel, 2001 ▸) revealed that the proteins share only a single identical residue, Glu138 in AtlE (shown in red), mutation of which to Ala rendered AtlE inactive, whereas the mutation of other acidic residues in the vicinity (Glu145, Asp167 and Asp227, which are 14, 11 and 8 Å away from the carboxylic O atoms of Glu138) exposed no additional residues assisting in catalysis. This latter indicated that the mechanism of catalysis is different from that observed in lysozymes (Vocadlo *et al.*, 2001 ▸). It merits mention that the alignment of the AtlE and LytB SP sequences starts at residues Asn82 and Asn130, respectively. The alignment of AtlE with the Lmo, FlgJ, ACOD and HLYZ sequences starts at AtlE residue Gly106 and the Gln78, A153, Ala42 and Lys1 residues of Lmo, FlgJ, ACOD and HLYZ, respectively. This indicates their large structural diversity in their N-terminal region corresponding to the R-lobe of AtlE.

All six helices comprising the core of AtlE (Figs. 4 ▸ and 5 ▸) are similar to the C-terminal domain of LytB SP, which the authors called the GH73 domain. The conservation of helices α6, α7, α12 and α14 shown in dark blue is typical for proteins that adopt the lysozyme-like fold. These helices form the central core of the AtlE structure and have counterparts in Lmo, FlgJ, HLYZ and ACOD (Fig. 5 ▸). The exceptions are the HLYZ structure, in which the C-terminal helix is broken into two parts (α6) and extended, and the α5 helix from the ACOD structure, which is curved, extended and wrapped along the inter-domain interface, from which the chain folds back and around the C-terminal helix and contacts the L-domain from below. The four-helical cores (three-helical in the cases of HLYZ and GLYZ) of the compared structures superimpose on the AtlE core with smaller r.m.s.d.s (1.1–3.5 Å) than the whole structures (Table 2 ▸), yet the similarity between the structures of lysozymes and AtlE is more at the level of the folding pattern than at the level of the structural details of amino-acid residues, apart from the catalytic Glu138.

The L-core is built similarly from α-helical elements in all compared structures (Fig. 5 ▸), whereas the R-cores differ in size and structure. The three helices from the L-core of AtlE shown in blue are present in all compared structures, whereas the AtlE α5 helix, shown in cyan, is present only in LytB SP. In the R-core, the AtlE C-terminal helix (α14), shown in blue, is present in all structures except HLYZ. In AtlE and ACOD the R-core is built from the N-terminal and C-terminal parts of the chain, whereas in the HLYZ, Lmo and FlgJ structures the R-core is folded entirely from the C-terminal part of the chain.

The R-lobe is unique to the AtlE structure and is absent in all others. It is built from the N-terminal parts of the sequence. In the LytB SP structure the GH73 domain does not have an R-lobe; its space is, however, occupied by the N-terminal domain. The L-lobes are present in all listed structures. They are mainly built from elements of β-structure, yet they differ in folding pattern and in their positioning. Only the HLYZ structure contains a three-stranded β-sheet, whereas in the Lmo and FlgJ structures there are long β-hairpins. β-Hairpins are also found in the AtlE structure. AtlE and LytB SP have an α-helix (AtlE has two) in this region.

Hence, the core regions share the four-helix core of the lysozyme fold; however, the L- and R-lobe regions responsible for substrate binding have little in common not only when comparing (mostly still putative) *N*-acetylglucosaminidases with lysozymes, but also among the structures and sequences of GH73 family members themselves (Figs. 5 ▸ and 6 ▸).

### 3.4. Substrate binding   

**Figure 7:**
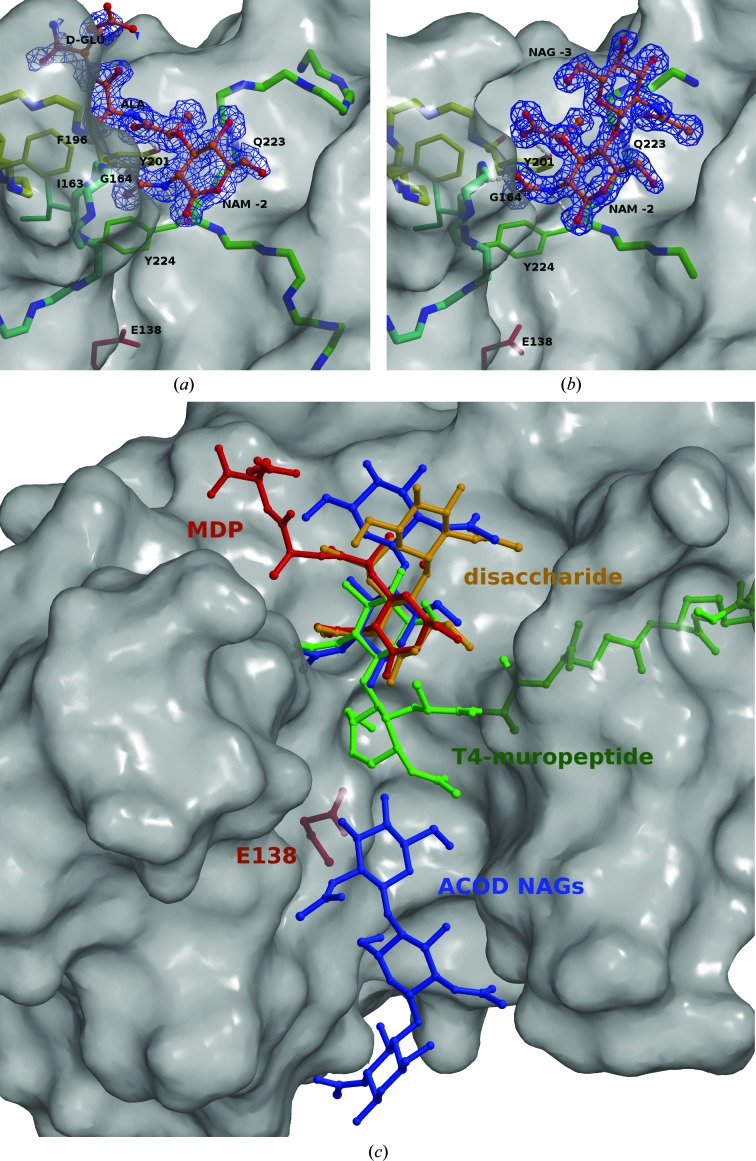
Complexes of AtlE with NAG-NAM and MDP. The AtlE structure is presented with a transparent surface, which makes the regions and residues in contact with the ligands visible. The averaged kick *F*_obs_ − *F*_calc_ electron-density map (Pražnikar *et al.*, 2009 ▸) contoured at 0.8σ and 1.2σ around the ligands is shown for (*a*) MDP and (*b*) the disaccharide NAG-NAM, respectively. The ligand residues and AtlE residues in contact with the ligands are marked, and their side chains are drawn in stick representation. The colours cyan, yellow and green indicates that the binding sites are built from three chain regions. The *N*-acetyl group of NAM is positioned equivalently in both complexes. Hydrogen bonds (grey dashed lines) pin NAM to the main-chain atoms of the Gly164 NH group and the Tyr224 carbonyl, while the O atoms of the lactyl moieties form a hydrogen bond to the OH group of the Tyr201 side chain. (*a*) The alanine hydrophobic side chain of MDP is positioned within the hydrophobic environment formed by the side chains of Ile163, Gly164 and Phe196, whereas the d-­Glu residue is disordered and points into the solvent, while (*b*) the *N*-acetyl group of the NAG residue forms a hydrogen bond to the main-chain NH group of Gln223. (*c*) Comparison of similar ligands superimposed on the AtlE structure. AtlE is shown as a transparent white surface with the catalytic Glu138 side chain labelled. The crystal structures of the muramyl dipeptide and the NAG-NAM disaccharide determined in complex with AtlE are shown as stick models in red and orange, respectively. They are labelled MDP and disaccharide. The muramyl dipeptide ligand bound to T4 lysozyme (T4_L; PDB entry 148l) is shown in green and labelled T4-muropeptide. The disaccharide and trisaccharide structures determined in complex with ACOD (PDB entry 3gxr) are shown in blue and labelled ACOD NAG. This figure was prepared with *MAIN* (Turk, 2013 ▸) and rendered with *Raster*3*D* (Merritt & Bacon, 1997 ▸).

To experimentally gain insight into the substrate-binding mechanism, we determined crystal structures of AtlE in complex with MDP (Fig. 7 ▸ *a*) and the NAG-NAM disaccharide (Fig. 7 ▸ *b*) synthesized as described in Fig. 3 ▸.

In the MDP complex the NAM and alanine residues are unambiguously resolved by the electron-density map, whereas the positioning of the atoms of the d-glutamic acid residue is less defined, as indicated by the electron-density map. The *N*-acetyl group of NAM is pinned to the surface of AtlE by hydrogen bonds to the main-chain atoms of the Gly164 NH group and the Tyr224 carbonyl (Fig. 7 ▸ *a*). The O atom of the lactyl moiety of the NAM residue forms a hydrogen bond to the OH group of the Tyr201 side chain. The alanine hydrophobic side chain is positioned within the hydrophobic environment formed by the side chains of Ile163, Gly164 and Phe196, whereas the d-glutamic acid residue is disordered and points into the solvent.

NAG-NAM is the smallest repeating unit of the glycan part of the peptidoglycan cell wall. In the complex of AtlE with NAG-NAM we observed that only a single molecule of the disaccharide was bound to the AtlE active site (Fig. 7 ▸ *b*). As the closest atom to the catalytic residue Glu138, the O1 atom of the NAM residue is positioned 6.7 Å away from the OE2 atom of the carboxylic group. The disaccharide is positioned above the Gln221–Ser226 loop shown in green. It is pinned to the surface at the bottom of the cleft by four hydrogen bonds: three formed by the NAM residue and one by NAG. The NAM moiety binds to the AtlE structure equivalently to that observed in the MDP–AtlE complex (Fig. 7 ▸ *a*). The *N*-acetyl group of the NAG residue forms a hydrogen bond to the main-chain NH group of Gln223. The *N*-acetyl group of NAM is pinned to the surface of AtlE by hydrogen bonds to the main-chain atoms of the Gly164 NH group and the Tyr224 carbonyl. The O atom of the lactyl moiety of the NAM residue forms a hydrogen bond to the OH group of the Tyr201 side chain. Numerous solvent molecules, two chloride ions and a sulfate ion are positioned in the region around the disaccharide.

To complement these structural data, we searched the PDB (Protein Data Bank; Berman *et al.*, 2000 ▸) for entries containing NAM residues (AMU according to the PDB nomenclature) and found several structures of NAM in complex with a hydrolase active site related to peptidoglycan substrate recognition. The structures were complexes of a NAM-peptide intermediate with T4 phage lysozyme (T4_L; PDB entry 148l; Kuroki *et al.*, 1993 ▸) and NAM-NAG-NAM in complex with chicken lysozyme (CLYZ; PDB entry 9lyz; Kelly *et al.*, 1979 ▸). Because the structural homology search showed similarity to the goose-type lysozyme from Atlantic cod (ACOD; PDB entry 3gxr; Helland *et al.*, 2009 ▸), we also included its complexes with NAG trimers. These ligand structures are shown superimposed on the AtlE structure in Fig. 7 ▸(*c*). The superimposed structures show similar positioning of the carbohydrate rings, yet different positions and orientations of the peptidyl extensions (the T4 muropeptide in green is pointing to the right and the AtlE-bound muropeptide MDP in red is pointing to the left), which provide insight into the difference in specificity between *N*-acetylgluco­s­aminidases and muramidases.

The five resolved NAG carbohydrate rings from the ACOD structure (PDB entry 3gxr; Helland *et al.*, 2009 ▸) fit into the active site of AtlE. A similar position is also occupied by NAG_3_ in complex with goose lysozyme (GLYZ; PDB entry 154l; Weaver *et al.*, 1995 ▸) and chicken lysozyme (CLYZ; PDB entry 9lyz; Kelly *et al.*, 1979 ▸). However, they are not shown in the figure because they overlap with the NAGs from the ACOD structure. Taken together, these structures show that carbohydrate rings are similarly positioned in all of these structures. They also reveal the positions of the subsites from −3 to +3 using the nomenclature proposed by Davies *et al.* (1997 ▸) or the B to G nomenclature as applied in the ACOD structural paper (Helland *et al.*, 2009 ▸). According to the Davies nomenclature, the observed NAM residues in the AtlE complexes (Figs. 7 ▸ *a* and 7 ▸ *b*) bind to the −2 sugar-binding subsite and NAG binds at the −3 subsite.

## 4. Discussion   

**Figure 8:**
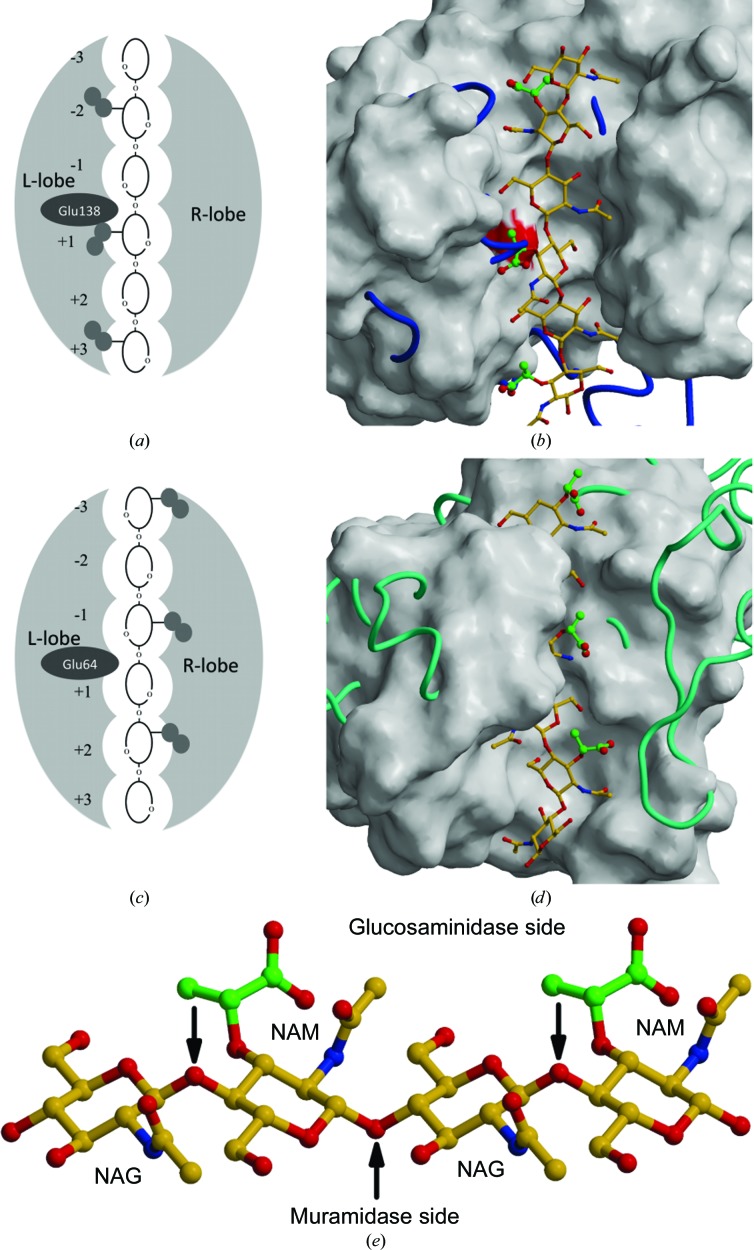
Structural differences between *N*-acetylglucos­aminidases and muramid­ases (lysozymes) in binding glycan cell-wall components. Images of three-dimensional models were prepared with *MAIN* (Turk, 2013 ▸) and rendered with *Raster*3*D* (Merritt & Bacon, 1997 ▸). (*a*) and (*c*) show a schematic representation of the approach of *N*-acetylglucosaminidases (*a*) and muramid­ases (*c*) to the poly-NAG-NAM saccharide, where the lactyl moieties are oriented towards the L- and R-lobes, which correspond to glucosaminidase and muramid­ase binding, respectively. (*b*) and (*d*) are models of the hexasaccharide (NAG-NAM)_3_ bound to the AtlE and ACOD active sites, shown in ball-and-stick representation against the surface of the targeted enzyme. The atom colour codes of the hexasaccharide model are blue and red for N and O atoms, respectively. C atoms are coloured orange, except for those from the lactyl group, which indicate the site of peptide-chain attachment; these are coloured green. The surface is white, except for the part corresponding to the carboxylic group of the catalytic residue Glu138, which is coloured red. The chain trace of ACOD is shown in blue against the surface of AtlE (*b*), whereas the chain trace of AtlE is shown in cyan against the surface of ACOD (*d*). (*e*) Cleavage sites of muramidases and *N*-acetylglucosaminidases. The three-dimensional model of the NAG-NAM-NAG-NAM tetrasaccharide is shown in ball-and-stick representation using the same colour codes as in (*b*) and (*d*). The cleavage sites of muramidases and *N*-acetylglucosaminidases are marked with arrows.

Using the gathered structural data, we addressed the substrate selectivity of the enzymes. We used the structures of the complexes shown in Fig. 7 ▸(*c*) as templates to generate models of hexasaccharides with an alternating NAG-NAM sequence in the structures of the active-site clefts of AtlE and ACOD as representative enzymes for the *N*-acetylglucosaminidase and muramidase activities, respectively. Fig. 8 ▸ shows a three-dimensional and schematic comparison of the bound substrate models. The chain trace of AtlE is shown on a background of the ACOD surface (Fig. 8 ▸ *d*) and *vice versa* (Fig. 8 ▸ *b*), while the substrate models correspond to the structures represented by surfaces. Figs. 8 ▸(*a*) and 8 ▸(*b*) and Figs. 8 ▸(*c*) and 8 ▸(*d*) demonstrate the differences between the shape of the active-site clefts and the way that the hexasaccharide substrates bind into them. Because the NAG and NAM residues are in alternating positions, the lactyl moieties shown in green are on the opposite sides of the active site in the AtlE (Fig. 8 ▸ *a*) and ACOD models (Fig. 8 ▸ *c*).

The opposite positioning of the lactyl moieties in the active sites of the *N*-acetylglucosaminidase AtlE and the muramid­ase ACOD predicts that features on the left side of the active-site cleft of AtlE are responsible for the recognition of lactyl moieties and peptides from the glycopeptides, whereas the features on the right side of the active-site cleft of AtlE should prevent the binding of lactyl moieties and peptides attached to them. The reverse is true for ACOD substrate binding. Indeed, Figs. 8 ▸(*a*) and 8 ▸(*b*) show that in the L-lobe and above the −2 and +1 positions of the lactyl moieties of NAM residues bound to the AtlE surface, there is sufficient space to accommodate the peptidyl extensions. However, in the ACOD structure there are features (shown in blue) protruding outside the AtlE surface that can prevent the binding of peptidyl extensions attached at these two positions. In accordance with Figs. 8 ▸(*c*) and 8 ▸(*d*), the reverse is true for the ACOD-bound substrate model. The AtlE hairpin region from Gly52 to Asn68 (shown in cyan) positioned at the top of the R-lobe of the AtlE structure (Fig. 8 ▸ *d*) forms the wall of the active site on the right and thereby prevents the binding of peptidyl extensions attached to the lactyl group of NAM residues, whereas the ACOD surface (Fig. 8 ▸ *d*) provides sufficient space to accommodate peptides bound to the lactyl groups of NAM residues. It should be mentioned that chicken-type lysozyme structures provide even fewer restraints than the goose type. This analysis demonstrates that the lobe regions in both types of enzymes indeed contain structural features that are responsible for the acceptance and rejection of the peptidyl moiety of the glycopeptide cell wall. Furthermore, the comparison of the chain trace of the lysozyme structures (ACOD, HLYZ and GLYZ), including the T4 phage lysozyme (T4_L), and *N*-acetylglucosaminidase structures (LytB SP, Lmo and FlgJ) superimposed on the AtlE structure in the regions of the lactyl moieties of the *N*-acetylglucos­aminidases revealed two common and relevant differences for substrate recognition. (i) The chains of lysozymes in the L-lobe region after the catalytic Glu run directly across the NAM 1 moiety, whereas in all compared *N*-acetyl­glucos­aminidase structures the chain after the catalytic site Glu folds to the left according to the view in Fig. 5 ▸. As consequence, the lysozyme and *N*-acetylgluco­saminidase loops building the L-lobe are positioned alternatively. (ii) The NAM −2 lactyl moiety is absent in chicken-type lysozyme structures (HLYZ, CLYZ and T4_L) owing to a different positioning of α12 (using the AtlE numbering). In goose-type lysozyme structures (GLYZ and ACOD) an additional helix, the last turn of which (Tyr151–Gly156 in ACOD) is followed by a loop, fills this space. These differences indicate that the analysis of the binding of substrate models to AtlE and ACOD is consistent with other structural data. (This latter analysis is not presented in a figure owing to the differences in the overlapping structural components which obscure the view, as indicated by the limited regions corresponding to equivalent parts and the rather large r.m.s.d. of their superimposition parameters shown in Table 2 ▸, and also by the low similarity at the sequence level shown in Fig. 6 ▸.) Hereby, we have answered the basic question as to how peptides attached to the peptido­glycan cell-wall component direct the docking to bring the desired glycosidic bond between the peptidyl NAM-NAG and NAG-NAM to the catalytic sites of *N*-acetylgluco­saminidases and lysozyme-like muramidases.

As our data and biochemical analysis of lysozyme activities (Vocadlo *et al.*, 2001 ▸) showed, selectivity between the NAG-NAM and NAM-NAG glycosidic bonds also exists at the level of the saccharide (NAG-NAM)_*n*_ and (NAG)_*n*_ substrates with no peptidyl extensions attached. In the substrate binding corresponding to muramidase activity the lactyl group is positioned in the −3, −1 and +2 subsites, whereas in the substrate binding corresponding to the *N*-acetylglucos­aminidase activity the lactyl group of *N*-acetylmuramic acid is positioned in the −2, +1 and +3 subsites. Clearly, there is no difference in the chemical environment of the glycosidic bonds between the two combinations of the carbohydrate rings, yet the muramidases cleave the glycosidic bond between the NAM O4 and NAG C1 atoms, whereas the *N*-acetyl­glucos­aminidases cleave the glycosidic bond between the NAG O4 and NAM C1 atoms. In addition, lysozymes/muramidases also cleave the glycosidic bond between two consecutive NAG residues, whereas we have shown here that AtlE and Glu-AtlA cannot. Evidently, the difference between the NAM residues and the NAG residues should come from recognition of the lactyl group.

Therefore, we searched for the structural features that are responsible for the acceptance and rejection of the lactyl moieties of the NAM residues. In the lysozyme complexes, the lactyl moiety is not stabilized by any interaction with the underlying enzyme structure, whereas the N atom of the amide link of alanine in phage lysozyme is oriented against the main-chain carbonyl group of Gln105 (Anderson *et al.*, 1981 ▸; Helland *et al.*, 2009 ▸; Kelly *et al.*, 1979 ▸; Kuroki *et al.*, 1993 ▸; Weaver *et al.*, 1995 ▸). This positioning indicates that lysozymes select the side of NAG-NAM polymers by excluding the approach of the lactyl moiety from the ‘wrong’ side, but do not require it at the other side. It also explains why lysozymes can also cleave NAG polymers. The AtlE–NAG-NAM complex structure presented here, however, reveals that the lactate group of the NAM −2 residue forms a hydrogen bond to Tyr201 and leaves sufficient space behind it to accommodate the peptidyl moiety (Figs. 7 ▸ *a* and 7 ▸ *b*). The recognition of the NAM residue leads to a twist in the NAG-NAM chain at the −3 position. The absence of AtlE activity against NAG substrates can be attributed to the extended but not twisted conformation of the NAG substrate, which disables productive binding at the −3 and −2 positions. This brief analysis indicates that *N*-acetylglucosaminidases direct the binding of polysaccharide NAG-NAM substrates by selective recognition of the lactyl moiety specific to the murein structure, whereas muramidases do not. This conclusion reveals an irony in the nomenclature introduced in the early days of NAG-NAM polysaccharide-degradation studies (Berger & Weiser, 1957 ▸), predating the structural insight available now. If history could be changed, this structural analysis would suggest that it may be more appropriate to swap the terms referring to the muramidase and *N*-acetylglucosaminidase activities, as only the latter is based on muramyl residue selection and binding, whereas the former does not require it.

Taken together, the analysis of structures of the AtlE and lysozyme complexes and saccharides enabled us to expose specific structural features that exclude the binding of the substrate molecules in an incompatible manner and thereby explain the difference between the *N*-acetylglucosaminidase and muramidase activities. To achieve this specificity, both enzyme families adapted to their respective target in the glycan substrate structure: the glycans linked with β-glycosidic bonds form extended structures with carbohydrate rings in the chair conformation. As shown in the side view (Fig. 8 ▸ *e*), the chain exhibits a zigzag pattern. The odd number of bonds (five) along the polysaccharide chain separating the two consecutive glycosidic bond O atoms positions the O atoms in alternating positions, where every other atom points either up or down. If the *N*-acetylglucosaminidases recognized the muramic moieties on the same side as the muramidases, then the catalytic residue from the bottom would not be able to reach the O atom of the glycosidic bond positioned at the top. Such binding would require approach of the catalytic residue from the top. To preserve the common catalytic residue construct, the glycan chain must be approached from two opposite sides and the substrate-selection mechanism is adopted for each case. Evolution has endowed *N*-acetyl­glucosaminidases with structural features that accept lactate moieties on NAM residues on the L-side of the active-site cleft, whereas muramidases achieve their specificity by not allowing them to bind on the L-side. The absence of selective recognition of the lactyl group on the R-side, however, enables them to process NAG polymers as well. As exposed by our structural analysis, this important difference in access to the active-site cleft suggests that *N*-acetylglucosaminidases may be suitable targets for novel antibiotic-discovery research. The extent of structural differences in the lobe regions among the *N*-acetylglucosaminidases, however, suggests that targeting of various bacterial species may require the design of species-specific drugs. If successful, such an approach may lead to diminished ‘pollution’ of the biosphere by reducing the harmful impact of the undesired spread of resistance against antibiotics and maintaining the normal microbiome (Blaser, 2016 ▸).

## Supplementary Material

PDB reference: AtlE, 4pia

PDB reference: complex with NAG-NAM, 4pi7

PDB reference: complex with MDP, 4pi9

PDB reference: E138A mutant, complex with NAG-NAM, 4pi8

## Figures and Tables

**Table 1:** Data-collection and refinement statistics for AtlE

Structure	SeMet	Native	NAG-NAM complex	E138A mutant, NAG-NAM complex	MDP complex
PDB code	4pia	4pia	4pi7	4pi8	4pi9
Crystal parameters					
Resolution range (Å)	34.1–1.40 (1.41–1.40)	23.00–1.47 (1.52–1.47)	50.00–1.60 (1.69–1.60)	38.40–1.39 (1.44–1.39)	38.75–1.48 (1.53–1.48)
Space group	*P*2_1_2_1_2_1_	*P*2_1_2_1_2_1_	*P*2_1_2_1_2_1_	*P*2_1_2_1_2_1_	*P*2_1_2_1_2_1_
Unit-cell parameters					
*a* (Å)	46.37	46.60	46.31	46.011	45.63
*b* (Å)	69.75	69.93	69.78	69.72	69.31
*c* (Å)	73.28	73.27	73.58	73.54	73.42
α = β = γ (°)	90	90	90	90	90
Data collection					
Beamline	Elettra XRD	Elettra XRD	Bruker Proteum	BESSY 14.1	BESSY 14.1
Wavelength (Å)	0.9786	1.0000	1.5410	0.9184	0.9184
Total reflections	405310	229540	222199	312334	251936
Unique reflections	46877 (1940)	41472 (3953)	31914 (2985)	48332 (4753)	39606 (3756)
Multiplicity	8.6 (4.2)	5.5 (3.7)	3.7 (1.9)	6.5 (6.5)	6.4 (6.5)
Completeness (%)	98.3 (82.7)	99.30 (95.97)	99.45 (94.82)	99.92 (99.69)	99.55 (96.26)
Mean *I*/σ(*I*)	15.4 (1.0)	39.2 (4.3)	24.24 (3.34)	26.91 (3.00)	20.16 (2.07)
Wilson *B* factor (Å^2^)		11.72	16.14	15.96	19.66
*R*_merge_	0.113 (0.884)	0.043 (0.259)	0.121 (0.234)	0.034 (0.593)	0.045 (0.746)
Refinement statistics					
*R*_work_		0.1492	0.1563	0.152	0.1772
*R*_free_		0.1715	0.1868	0.1755	0.208
No. of non-H atoms					
Total		2111	2116	2146	2111
Macromolecules		1844	1826	1837	1832
Ligands		9	50	53	42
Water		258	240	256	237
Protein residues		225	223	222	223
R.m.s.d., bonds (Å)		0.017	0.015	0.018	0.015
R.m.s.d., angles (°)		1.81	1.64	1.9	1.7
Ramachandran favoured (%)		97	97	97	98
Clashscore		2.45	3.24	2.94	2.45
Average *B* factors (Å^2^)					
Overall		16.6	21.7	23.2	29.5
Macromolecules		14.9	20.2	21.7	27.9
Ligands		22.1	22.5	21.4	39.0
Solvent		28.2	33.4	34.4	40.1

**Table 2:** Superimposition of structures Structures from the GH73 family and selected lysozymes were superimposed by 3*D_CE* (Shindyalov & Bourne, 1998 ▸) as a whole and in the region of the four conserved helices. The values for the latter are shown in parentheses. The columns indicate the structures, their PDB codes, their whole chain lengths, their superimposed residues, the r.m.s.d.s of their deviations and the identities of the residues in the superimposed regions.

Structure	PDB code	Chain length	Superimposed residues	R.m.s.d. (Å)	Chain identity (%)
LytB SP	4q2w	263	139 (61)	1.9 (1.1)	36 (38)
Lmo	3fi7	177	115 (53)	2.9 (1.3)	23 (21)
FlgJ	2zyc	156	118 (41)	3.2 (3.1)	25 (24)
ACOD	3gxk	185	124 (59)	4.7 (1.4)	9 (7)
HLYZ	1iwt	130	115 (33)	5.0 (2.2)	10 (9)
GLYZ	154l	185	122 (55)	3.7 (1.5)	10 (11)
T4_L	148l	162	96 (33)	4.7 (2.5)	5 (12)
CLYZ	9lyz	129	102 (23)	4.4 (3.5)	8 (4)
